# The Association of Morning Hypertension With Target Organ Damage in Patients With Chronic Kidney Disease and Hypertension

**DOI:** 10.3389/fcvm.2021.715491

**Published:** 2021-08-26

**Authors:** Xiang Liu, Fangming Li, Ting Zhang, Zhiyao Zheng, Huan Zhou, Aiya Qin, Yi Tang, Wei Qin

**Affiliations:** ^1^Division of Nephrology, Department of Medicine, West China Hospital, Sichuan University, Chengdu, China; ^2^West China School of Medicine, Sichuan University, Chengdu, China; ^3^Division of Nephrology, Department of Medicine, Chengdu Seventh People's Hospital, Chengdu, China

**Keywords:** morning hypertension, ambulatory blood pressure monitoring, chronic kidney disease, left ventricular mass index, urinary protein-creatinine ratio, estimated glomerular filtration rate

## Abstract

**Objectives:** To determine the association between morning hypertension and target organ damage (TOD) in patients with chronic kidney disease (CKD) and hypertension.

**Methods:** In this cross-sectional study, 447 patients with CKD and hypertension from two centers were enrolled. Ambulatory blood pressure monitoring was conducted in all patients. Linear regression and logistic regression analysis were used to determine the association between morning hypertension and TOD in patients with CKD and hypertension, including assessments of estimated glomerular filtration rate (eGFR), left ventricular mass index (LVMI), urine protein/creatinine ratio (UPCR), and left ventricular hypertrophy (LVH).

**Results:** Overall, 194 (43.4%) participants had morning hypertension. Morning hypertension was strongly correlated with LVH [odds ratio (OR), 2.14; 95% confidence interval (CI), 1.3–3.51; *p* < 0.01], lower level of eGFR (β = −0.51; 95%CI, −0.95–−0.08; *p* < 0.05), higher LVMI (β = 0.06; 95%CI, 0.04–0.08, *p* < 0.001), and UPCR (β = 0.22; 95%CI, 0.06–0.38, *p* < 0.01), independent of nocturnal hypertension and elevated morning blood pressure surge. As a continuous variable, both morning systolic blood pressure (SBP) and diastolic blood pressure (DBP) were found to be associated with LVH and higher level of UPCR and LVMI (*p* < 0.05), whereas only morning SBP was negatively correlated with eGFR (*p* < 0.01).

**Conclusion:** Morning hypertension was strongly correlated with cardiac damage and impaired kidney function in CKD patients with hypertension, independent of nocturnal hypertension and morning surge in blood pressure. Morning hypertension in CKD patients warrants further attention.

## Introduction

Hypertension is very common in patients with chronic kidney disease (CKD), with the prevalence of 67–92% (1); because blood pressure (BP) measured using ambulatory blood pressure monitoring (ABPM) strongly associated cardiovascular (CV) events and renal outcomes, ABPM is considered the preferred metric of BP in both the general population and CKD patients (2). Furthermore, compared with office BP, ABPM is a better predictor of hypertension-mediated organ damage (3) and a more sensitive risk factor for CV events and mortality (4, 5). Therefore, according to current guidelines, ABPM is recommended for application in clinical practice (6).

Morning hypertension has been a recent research focus. It was shown to be associated with target organ damage (TOD) including left ventricular mass index (LVMI), urine albumin/creatinine ratio, maximum carotid intima media thickness (7, 8), and CV events (9, 10) in general or hypertensive patients. Experts from Asia suggested that the measurement and treatment of morning hypertension should be an indispensable part of treatment for hypertensive patients (11). However, studies on the association between morning hypertension and TOD in CKD patients are scarce. It is therefore imperative to investigate the prevalence and role of morning hypertension in CKD patients with TOD, given the high risk of progression to end-stage renal disease and CV damage in these patients.

Morning hypertension is consistently accompanied by nocturnal hypertension or elevated morning surge in blood pressure, which are also reported to be associated with TOD (12, 13). Therefore, some researchers suggested that the association between morning hypertension and TOD may be attributed to nocturnal hypertension or morning blood pressure surge (14, 15). In a recent cross-sectional study, Ye et al. demonstrated that the impact of morning hypertension on LVMI is dependent on the morning surge in normotensive patients (8). Additionally, a study conducted by Oh et al. found that only morning hypertension combined with elevated nocturnal hypertension was associated with vascular organ damage and high central BP (16).

We conducted the present cross-sectional study to further our understanding of the association between morning hypertension and TOD in patients with CKD and hypertension and to determine whether this association is independent of nocturnal hypertension and the morning surge in blood pressure.

## Materials and Methods

### Participants

This cross-sectional study included patients from two centers (West China Hospital and Chengdu Seventh People's Hospital) in China. Adult patients were eligible if they (1) had CKD, (2) were diagnosed with hypertension, and (3) agreed to undergo 24 h ABPM. Exclusion criteria were patients (1) on dialysis (hemodialysis or peritoneal dialysis), (2) with history of malignancy, (3) with <70% valid records on 24 h ABPM, and (4) who were pregnant. Patients with albuminuria (albumin/creatinine ratio ≥30 mg/g) or estimated glomerular filtration rate (eGFR) <60 ml/min/1.73 m^2^ or abnormalities of kidney structure for over 3 months were diagnosed with CKD (17). eGFR was determined from serum creatinine levels using the Chronic Kidney Disease Epidemiology Collaboration (CKD-EPI) equation (18). The study protocol was approved by the ethics committee of the West China Hospital, Sichuan University, and was approved by the Institutional Review Board. Written informed consent was obtained from all patients.

A total of 447 CKD patients formed the cohort of this cross-sectional study. Baseline evaluation including medical history, physical examination, and laboratory tests were recorded at the time patients visited the outpatient clinic or were admitted to hospital. These data included sex, age, body mass index (BMI), current alcohol consumption, current smoking, CV history, antihypertensive drugs, diabetes mellitus (DM), biochemical parameters, urinary protein test, and two-dimensional echocardiogram.

### Blood Pressure Measurements

Experienced nurses measured office BP when participants were admitted to our hospital or visited the outpatient clinic. After patients were allowed to rest quietly for 5–10 min, the mean value of three consecutive BP measurements at 5-min intervals using a mercury sphygmomanometer was recorded. The 24-h ABPM was performed using Space labs 90217 devices (Space labs Medical, Redmond, WA, USA) at West China Hospital and an ABPM 6100 (Welch Allyn, Chicago, IL, USA) at Chengdu Seventh People's Hospital, with BP readings set at 20-min intervals from 6:00 a.m. to 10:00 p.m., and 30- or 60-min intervals from 10:00 p.m. to 6:00 a.m. Day- and nighttime BPs were defined as mean BP during the period from 6:00 a.m. to 10:00 p.m. and 10:00 p.m. to 6:00 a.m., respectively. Patients were instructed to undergo their usual activities and take antihypertensive drugs as usual and were encouraged to sleep no later than 10:00 p.m. and to get up at approximately 6:00 a.m. ABPM was not conducted if the patient worked nightshifts. The time patients went to bed and woke up was recorded. A measurement with at least 70% of diurnal and nocturnal BP readings was regarded as a successful ABP. The ABP must be taken within 3 days after the measurement of office BP. Both office BP and ABP measurements were taken from the non-dominant arm with an appropriate cuff size based on arm circumference at the time of enrollment.

### Cardiac Assessment

Echocardiography was performed by two experienced ultrasonologists according to standardized procedures, as previously reported (19). The linear method was applied to quantify LV mass (20): LVM (g) = 0.8 × 1.04 × [(interventricular septum + LV internal diameter + posterior wall thickness)^3^ – LV internal diameter^3^] + 0.6 g. Left ventricular hypertrophy (LVH) was defined according to the 2015 American Society of Echocardiography/European Association of Cardiovascular Imaging chamber quantification document, with LVMI per body surface area >95g/m^2^ in women and 115 g/m^2^ in men (19).

### Definitions

Morning hypertension was defined as mean BP ≥135/85 within 2 h after waking (11). Morning surge in blood pressure was defined as SBP during the 2-h period immediately after waking minus the average of three SBP readings centered around the lowest nighttime SBP value (21). Patients were divided into the elevated morning surge group (≥15 mmHg) and low morning surge group (<15 mmHg) according to the third quantile. Dipping patterns of BP and heart rate (HR) were calculated according to the following formula: mean night/day ratio of SBP, DBP, and HR. Patients were diagnosed as dippers if the ratio was >0.8–0.9, non-dippers if the ratio was >0.9–1, or reverse dippers if the ratio was >1 (22). Patients were defined to have achieved the goal for ambulatory BP when 24-h, daytime, and nighttime BP was <130/80, <135/85, and <120/70 mmHg, respectively, and to have the goal for office BP when the BP was <140/90 (23). CV history was defined as history of heart disease, peripheral vascular disease, or cerebral vascular disease.

### Statistical Analyses

Statistical analyses were performed using IBM SPSS Statistics for Windows, Version 20.0 (IBM Corp, Armonk, NY, USA) and GraphPad Prism 8.0.1. Normally distributed data are presented as mean ± standard deviation, skewed data as median values with interquartile range, and categorical variables as numbers with percentage. Log-transformation or square root calculations were applied for normal transformation. Data were analyzed using the chi-square test or Fisher's exact test for categorical variables, the Student's *t*-test for normally distributed data, and the Wilcoxon rank-sum test for continuous skewed variables.

Receiver-operating characteristic (ROC) curve analysis was conducted, and the area under the curve (AUROC) was calculated to assess the ability of night blood pressure and morning blood pressure surge to predict morning hypertension. Binary logistic regression was applied to detect the factors associated with morning hypertension and to calculate the risk of LVH correlated with morning hypertension. Linear regression analysis was used to identify the association between morning hypertension and eGFR, UPCR, and LVMI. The regression β-coefficient represented the contribution of the independent variables to the dependent variables. To determine whether the correlation between morning hypertension and TOD was independent of night hypertension or morning surge, we added night hypertension and morning surge to the regression model in models 2 and 3, respectively. Odds ratios (ORs) and 95% confidence intervals (CIs) were calculated, and a two-tailed *p* < 0.05 was considered statistically significant.

## Result

### Baseline Features

In total, 583 patients were enrolled. A total of 136 were excluded because of the following factors: complication with malignant tumors (5), incomplete ABPM data (50), and dialysis (81). Finally, 447 patients with CKD and hypertension were included in this study. Two hundred (44.7%) patients were male, and the mean age was 67 ± 14 years. A total of 194 (43.4%) participants had morning hypertension, among which 186 (95.9%) had nocturnal hypertension, and 97 (50%) had morning surge in blood pressure with ≥15 mmHg. A total of 320 (71.6%) participants had nocturnal hypertension, and the morning surge in 152 (34%) patients was ≥15 mmHg.

Baseline features of the participants grouped according to morning BP are shown in Table 1. No differences in age, sex, current smoking, current alcohol consumption, CV history, or DM was found between the two groups. Compared with those with morning normotension, the proportion of use of calcium channel blockers, α blockers, and over two kinds of antihypertensive drugs was higher in patients with morning hypertension. Higher levels of white blood cells, neutrophils, creatinine, uric acid, and phosphorus were observed in the morning hypertension group, whereas the levels of lymphocytes, total protein, albumin, and calcium were lower in this group (Table 1). Regarding the BP parameters, control of 24 h, daytime, and nighttime BP and that measured at the clinic were all poorer in the morning hypertension group (*p* < 0.001). No differences were found in SBP, DBP, or HR dipping between the two groups (Table 2).

**Table 1:** Demographic and clinical characteristics between morning hypertension and morning normotension groups.

**Variables**	**Morning** **normotension** **(*n* = 253)**	**Morning** **hypertension** **(*n* = 194)**	***P***
Age	72 (59, 79)	68 (56, 78)	NS
Gender (male, n, %)	112 (44.3)	88 (45.4)	NS
BMI	23.88 (22.23, 26.26)	24 (22.49, 26.72)	NS
Smoking	39 (15.4)	29 (14.9)	NS
Alcohol	36 (14.2)	12.4	NS
CV history	83 (32.8)	62 (32)	NS
DM	154 (60.9)	123 (63.4)	NS
CCB	137 (54.2)	146 (75.3)	<0.001
RAS	114 (45.1)	96 (49.5)	NS
Diuretic	30 (11.9)	29 (14.9)	NS
β blocker	72 (28.5)	72 (37.1)	NS
α blocker	6 (2.4)	20 (10.3)	<0.001
Numbers of drugs (>2)	41 (16.2)	50 (25.8)	0.013
Biomarkers			
Red blood cells	4.05 (0.70)	4.01 (0.90)	NS
Hemoglobin	120.03 (21.37)	116.85 (24.90)	NS
White blood cells	6.15 (4.74, 7.61)	6.69 (5.44, 8.42)	0.003
Neutrophil	0.65 (0.12)	0.68 (0.11)	0.007
Lymphocyte	0.24 (0.11)	0.22 (0.10)	0.049
Total protein	68.44 (6.99)	66.71 (9.91)	0.031
Albumin	39.50 (5.27)	37.62 (6.89)	0.001
Creatinine	111.8 (88.95, 143.15)	126.3 (95.95, 182.3)	0.001
Uric acid	344.36 (278, 435)	374 (311.75, 456.75)	0.005
Glucose	6.5 (5.2, 8.8)	6.49 (5.27, 9.44)	NS
Potassium	**4.07 (0.55)**	**4.08 (0.60)**	**NS**
Sodium	140.1 (137.7, 142.5)	140.20 (137.70, 142.60)	NS
Chlorine	105.93 (4.95)	105.92 (5.52)	NS
Calcium	2.30 (2.18, 2.43)	2.27 (2.12, 2.41)	0.036
Phosphorus	1.04 (0.92, 1.22)	1.14 (0.97, 1.29)	<0.001
Magnesium	0.86 (0.80, 0.93)	0.85 (0.80, 0.93)	NS
Cholesterol	4.31 (3.60, 5.18)	4.49 (3.70, 5.34)	NS
Triglyceride	1.49 (0.96, 2.23)	1.52 (1.08, 2.41)	NS
High density lipoprotein	1.18 (0.96, 1.44)	1.17 (0.92, 1.52)	NS
Low density lipoprotein	2.72 (2.02, 3.43)	2.60 (1.93, 3.43)	NS

*BMI, body mass index; CV, cardiovascular; DM, diabetes mellitus; CCB, calcium channel blocker; RAS, renin–angiotensin system*.

**Table 2:** The blood pressure characteristics from ABPM between morning hypertension and morning normotension groups.

**Variables**	**Morning** **normotension** **(*n* = 253)**	**Morning** **hypertension** **(*n* = 194)**	***p***
Office SBP	138 (124, 148)	145 (134, 160)	<0.001
Office DBP	78 (70, 85)	81.5 (73.5, 92)	<0.001
Office BP control	129 (51)	61 (31.4)	<0.001
**24 h**			
SBP	120.32 (11.72)	142.08 (13.69)	<0.001
DBP	65.53 (9.00)	76.41 (12.72)	<0.001
Heart rate	72 (10)	75 (11)	0.047
24 h BP control	195 (77.1)	24 (12.4)	<0.001
**Day**			
SBP	121.02 (12.08)	142.75 (13.60)	<0.001
DBP	66.47 (9.58)	77.20 (13.19)	<0.001
Heart rate	74 (10)	76.44 (11.98)	0.015
Day BP control	215 (85)	42 (21.6)	<0.001
**Night**			
SBP	118.74 (13.19)	140.79 (17.02)	<0.001
DBP	63.41 (9)	74.48 (12.83)	<0.001
Heart rate	65 (61, 73)	69 (62.75, 76)	0.01
Night BP control	119 (47)	8 (4.1)	<0.001
SBP dipping	0.98 (0.07)	0.99 (0.08)	NS
DBP dipping	0.96 (0.08)	0.97 (0.09)	NS
Heart rate dipping	0.92 (0.08)	0.92 (0.08)	NS
Morning surge in blood pressure	6.21 (12.38)	15.74 (14.98)	<0.001
Elevated morning surge in blood pressure	55 (21.7)	97 (50)	<0.001
ABP control	118 (46.6)	7 (3.6)	<0.001
Morning SBP	118.74 (10.48)	149.63 (14.64)	<0.001
Morning DBP	66.26 (9.49)	81.21 (14.50)	<0.001

*SBP, systolic blood pressure; DBP, diastolic blood pressure; BP, blood pressure; ABP, ambulatory blood pressure*.

Association between nighttime blood pressure and morning surge in blood pressure with morning hypertension.

Among patients in the morning hypertension group, only 4.1% had normal nighttime BP, compared with 47% in the morning normotension group (*p* < 0.001). Moreover, 97 (50%) patients with morning hypertension had elevated morning surge, while the rate was 21.7% in patients with morning normotension (*p* < 0.001) (Table 2). According to the multivariate logistic regression model, nighttime SBP (OR, 1.2; 95%CI, 1.11–1.3) and morning surge (OR, 1.2; 95%CI, 1.15–1.26) were independently correlated with morning hypertension (*p* < 0.001) (Table 3).

**Table 3:** Binary logistic regression analysis for factors associated with morning hypertension.

**Variables**	**OR (95% ci)**	**p**
Night SBP	1.20 (1.11, 1.30)	<0.001
Night DBP	1.02 (0.92, 1.14)	NS
Day SBP	1.02 (0.95, 1.10)	NS
Day DBP	1.07 (0.95, 1.20)	NS
Morning surge in blood pressure	1,20 (1.15, 1.26)	<0.001

*SBP, systolic blood pressure; DBP, diastolic blood pressure; BP, blood pressure*.

**Figure 1:**
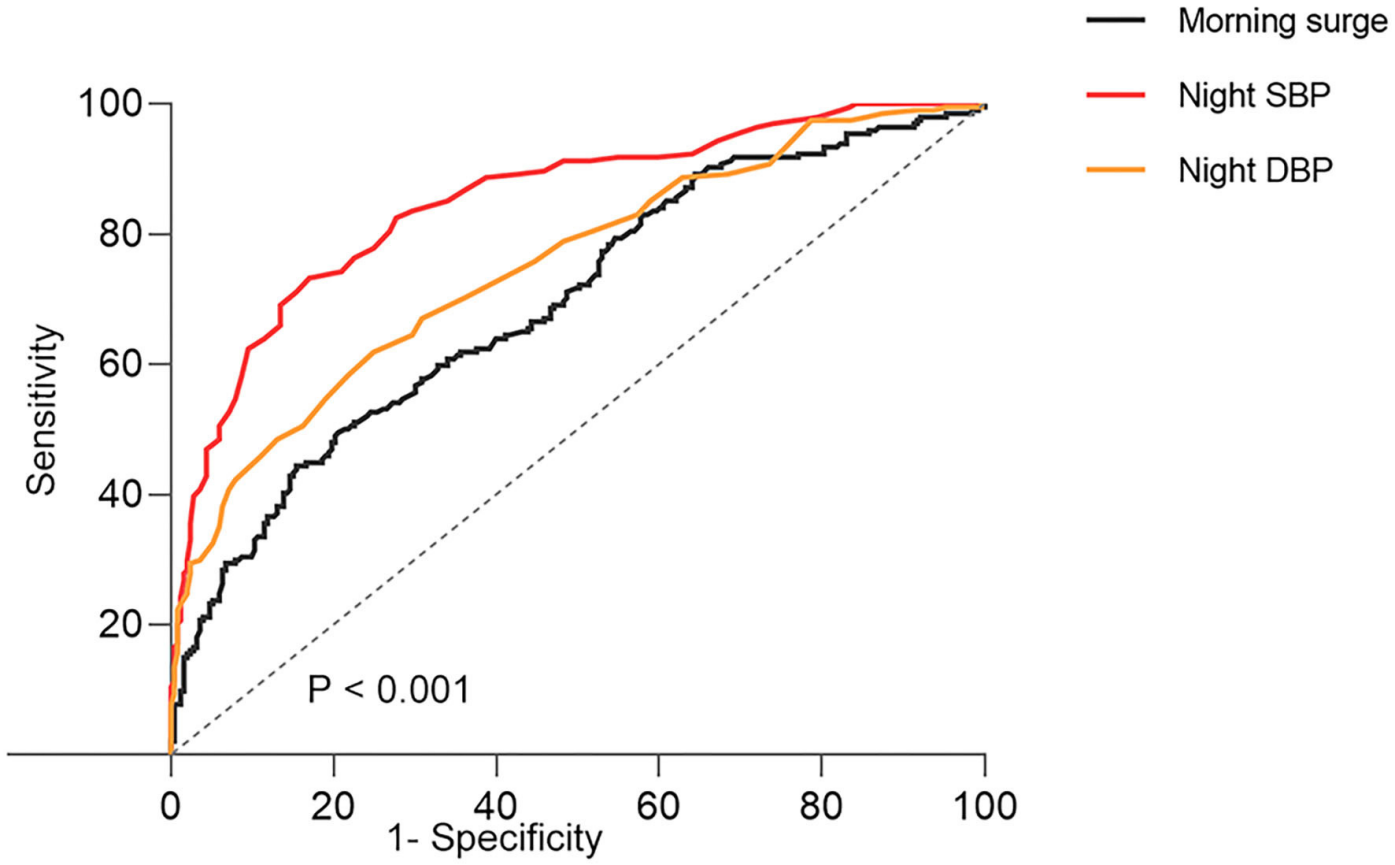
The ROC curves of morning surge in blood pressure and night blood pressure about morning hypertension. SBP, systolic blood pressure; DBP, diastolic blood pressure; ROC, receiver-operating characteristic.

To assess the ability to use nighttime BP and morning surge in blood pressure for predicting morning hypertension, ROC curve analysis was performed (Figure 1). Nighttime SBP could better distinguish morning hypertension (AUROC, 0.85; 95%CI, 0.81–0.88; *p* < 0.001), compared with nighttime DBP (AUROC, 0.75; 95%CI, 0.71–0.88; *p* < 0.001) and morning surge (AUROC, 0.69; 95%CI, 0.65–0.74; *p* < 0.001). The optimal cutoff points for morning hypertension were 130 mmHg for nighttime SBP (sensitivity, 73.2%; specificity, 83%) and 70 mmHg for nighttime DBP (sensitivity, 62%; specificity, 75%).

### Association Between Morning Hypertension and Target Organ Damage

According to univariate analysis, both morning hypertension and nocturnal hypertension were shown to be associated with higher level of LVMI and UPCR, lower eGFR (p < 0.01), and LVH (*p* < 0.001), whereas only higher level of UPCR was found to correlate with elevated morning surge in blood pressure (*p* = 0.026) (Supplementary Tables 1–3).

According to multivariate linear and logistic regression analysis, morning hypertension correlated strongly with lower level of eGFR (β = −0.51; 95%CI, −0.95–−0.08; *p* < 0.05), higher level of LVMI (β = 0.06; 95%CI, 0.04–0.08; *p* < 0.001), UPCR (β = 0.22; 95%CI, 0.06–0.38; *p* < 0.01), and LVH (OR, 2.14; 95%CI, 1.3–3.51; *p* < 0.01) when nocturnal hypertension and elevated morning surge in blood pressure were considered (Table 4, model 3). After further adjustments of baseline features (age, sex, BMI, current alcohol consumption, current smoking, DM, and CV history), biochemical indices, and office BP control, the significance did not change. When morning hypertension was considered, TOD was not shown to be associated with nocturnal hypertension and elevated morning surge in blood pressure, except for UPCR, which was higher in patients with nocturnal hypertension (Table 4). However, the difference was not significant after adjustment of biochemical indices and office BP.

**Table 4:** Multivariate linear regression and binary logistic regression analysis of BP indexes and target organ damage.

**Variables**	**Model 1**	**Model 2**	**Model 3**	**Model 4**	**Model 5**
$\sqrt{eGFR}$					
Morning hypertension	−0.58 (−0.94, −0.22)^#^	−0.44 (−0.85, −0.03)^a^	−0.51 (−0.95, −0.08)^a^	−0.52 (−0.95, −0.09)^a^	−0.47 (−0.83, −0.11)^#^
Nocturnal hypertension	–	−0.33 (−0.78, 0.12)	−0.29 (−0.75, 0.17)	−0.39 (−0.84, 0.07)	−0.16 (−0.54, 0.21)
Elevated morning surge in blood pressure	–	–	0.19 (−0.21, 0.59)	0.09 (−0.31, 0.49)	0.06 (−0.27, 0.39)
**Lg LVMI**					
Morning hypertension	0.06 (0.05, 0.08)^*^	0.05 (0.03, 0.07)^*^	0.06 (0.04, 0.08)^*^	0.06 (0.04, 0.08)^*^	0.05 (0.03, 0.07)^*^
Nocturnal hypertension	–	0.03 (0.002, 0.05)^a^	0.02 (−0.002, 0.05)	0.02 (−0.001, 0.05)	0.02 (−0.01, 0.04)
Elevated morning surge in blood pressure	–	–	−0.02 (−0.04, 0.004)	−0.02 (−0.04, 0.01)	−0.01 (−0.03, 0.01)
**Lg UPCR**					
Morning hypertension	0.35 (0.22, 0.48)^*^	0.26 (0.12, 0.41)^*^	0.22 (0.06, 0.38)^#^	0.22 (0.07, 0.38)^#^	0.15 (0.02, 0.29)^a^
Nocturnal hypertension	–	0.21 (0.04, 0.37)^#^	0.23 (0.07, 0.40)^#^	0.22 (0.06, 0.38)^#^	0.11 (−0.03, 0.25)
Elevated morning surge in blood pressure	–	–	0.11 (−0.04, 0.26)	0.10 (−0.05, 0.25)	0.08 (−0.05, 0.20)
**LVH**					
Morning hypertension	2.59 (1.71, 3.92)^*^	2.08 (1.31, 3.31)^#^	2.14 (1.30, 3.51)^#^	2.13 (1.26, 3.61)^#^	1.86 (1.08, 3.26)^a^
Nocturnal hypertension	–	1.74 (0.98, 3.10)	1.72 (0.96, 3.07)	1.97 (1.06, 3.65)^a^	1.73 (0.91, 3.26)
Elevated morning surge in blood pressure	–	–	0.93 (0.59, 1.48)	1.06 (0.65, 1.74)	1.20 (0.72, 1.99)

* *p < 0.001;*

# *p < 0.01;*

a p < 0.05. Model 1: morning hypertension; model 2: model 1 + nocturnal hypertension; Model 3: model 2 + morning surge in blood pressure; Model 4: model 3 + age, sex, BMI, alcohol, smoking, CV history, diabetes mellitus; Model 5: model 4 + hemoglobin, albumin, sodium, potassium, triglyceride, office BP control for eGFR; hemoglobin, albumin, sodium, potassium, creatinine, uric acid, triglyceride, office BP control for Lg LVMI, hemoglobin, albumin, glucose, p^*^ca, creatinine, cholesterol, high-density lipoprotein, low-density lipoprotein, office BP control for Lg PCR; hemoglobin, albumin, creatinine, sodium, potassium, triglyceride, high-density lipoprotein, low-density lipoprotein, office BP control for LVH. LVMI, left ventricular mass index; UPCR, urine protein–creatinine ratio; eGFR, estimated glomerular filtration rate.

**Figure 2:**
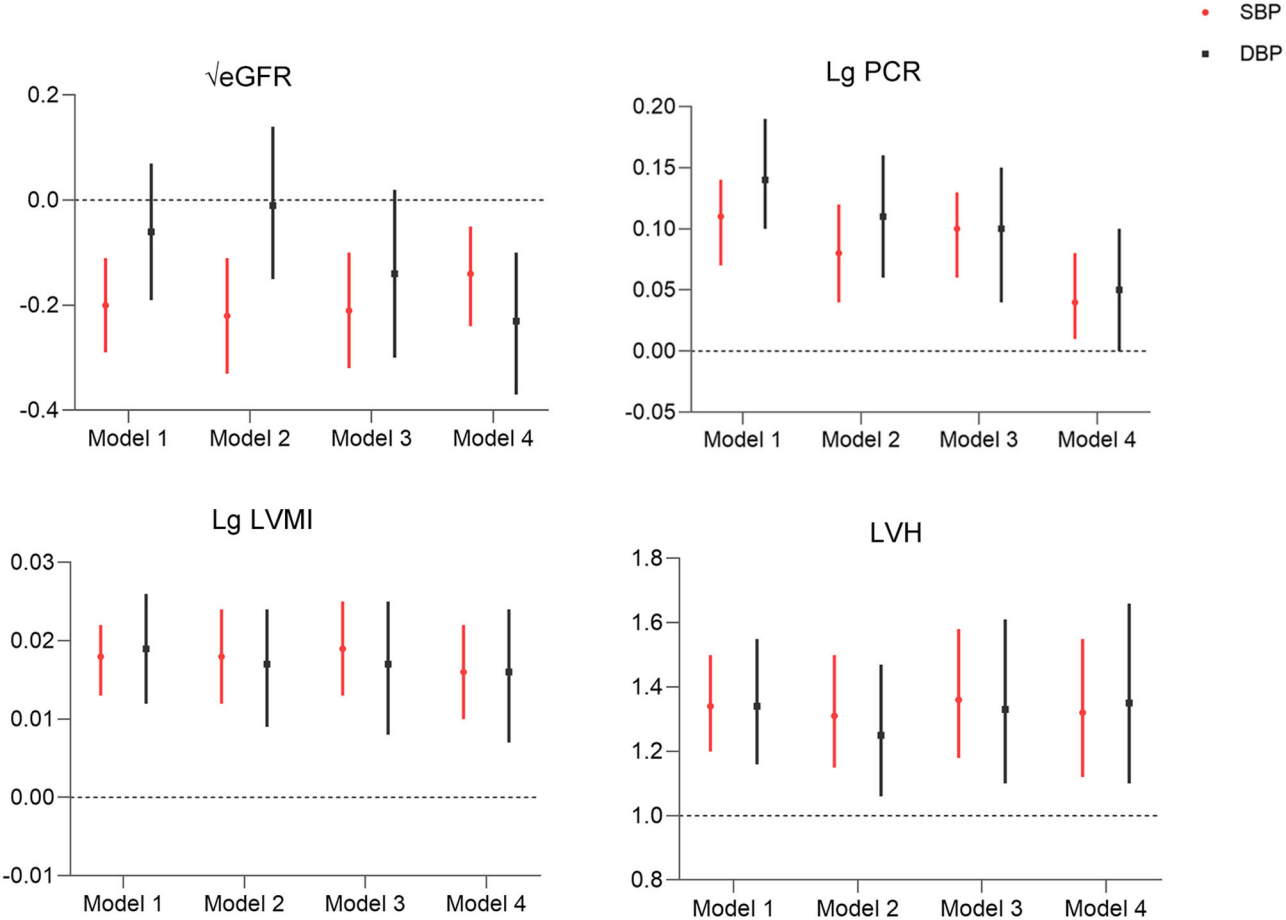
Association of morning systolic blood pressure and diastolic blood pressure (per 10 mmHg) with target organ damage. Model 1: morning hypertension; model 2: model 1 + nocturnal hypertension + morning surge in blood pressure; model 3: model 2 + age, sex, BMI, alcohol, smoking, CV history, DM; model 4: model 3 + hemoglobin, albumin, Na, k, TG, office BP control for eGFR; hemoglobin, albumin, Na, K, creatinine, uric acid, TG, office BP control for Lg LVMI, hemoglobin, albumin, glucose, p*ca, creatinine, CHOL, HDL, LDL, office BP control for Lg PCR; hemoglobin, albumin, Creatinine, Na, K, TG, HDL, LDL, office BP control for LVH. LVMI, left ventricular mass index; PCR, protein–creatinine ratio; eGFR, estimated glomerular filtration rate; LVH, left ventricular hypertrophy.

To further assess the correlation between morning BP and TOD, morning SBP and DBP were analyzed as continuous variables in the regression model. Both morning SBP and DBP were found to be associated with LVH, higher UPCR, and LVMI (Figure 2, Supplemental Table 4). Regarding eGFR, only morning SBP was shown to be negatively correlated with eGFR (all *p* < 0.01), and the correlation between morning DBP and eGFR was only significant in model 4 (Figure 2, Supplementary Table 4).

## Discussion

In this cross-sectional study, we found that the prevalence of morning hypertension in CKD patients was 43.4 and 95.9% of these patients also had nocturnal hypertension. Morning hypertension was primarily determined by nighttime BP and morning surge in blood pressure, and nighttime SBP distinguished morning hypertension effectively (AUROC, 0.85; 95%CI, 0.81–0.88; *p* < 0.001). Morning hypertension was strongly correlated with TOD in CKD patients, including cardiac damage (LVH, higher level of LVMI) and kidney function decline (higher UPCR and lower eGFR), independent of nocturnal hypertension and morning surge in blood pressure. As a continuous variable, both morning SBP and DBP correlated with UPCR, LVMI, and LVH, whereas only morning SBP was negatively associated with eGFR. These data suggest that morning hypertension plays an important role in TOD in patients with CKD and hypertension.

The rate of morning hypertension reached 43.4% in this study, and the prevalence varies substantially across countries and populations (from 15.9 to 60.7%) (24). Our study validated that morning hypertension can result from increased nocturnal BP and large morning surge in blood pressure. In this study, 95.9% of patients with morning hypertension also had nocturnal hypertension, and nighttime SBP could well distinguish morning hypertension. This indicated the strong association between nocturnal BP and morning BP in patients with CKD and hypertension.

Previous studies showed that morning hypertension is associated with higher risk of LVH (8), which is a strong predictor of poor CV and renal outcomes in both CKD and general patients (25, 26). This may be a factor explaining why morning hypertension was found to be associated with CV events in some prospective studies (9, 10). However, studies on the association between morning hypertension and CV events in CKD patients are very limited. According to a recent cross-sectional study, only masked morning hypertension was demonstrated to be associated with increased prevalence of LVH in CKD patients (27). Our study further confirmed this observation. Both morning SBP and DBP correlated strongly with LVH and LVMI. The strong correlation may be supported by the observation that the high awaking BP was used to detect growth of LVM over time in CKD patients (28).

Our study showed that morning hypertension was associated with increased prevalence of kidney function decline, including higher UPCR and lower eGFR. Similar findings were reported in some previous studies (7, 14, 15). Hypertension may result in endothelial dysfunction, which subsequently contributes to increased arterial stiffness. This in turn can facilitate escape of albumin from renal glomeruli (29, 30). Interestingly, only morning SBP, not DBP, correlated with eGFR decline, which was consistent with previous studies (14, 15). However, the underlying mechanisms are not clearly defined. Previous studies suggested that the association between morning hypertension and TOD may be attributed to nocturnal hypertension or morning surge in blood pressure (14, 15). However, in our study, after adjustment of nocturnal hypertension and morning surge, morning hypertension showed an attenuated but nonetheless strong correlation with TOD, indicating that the correlation between morning hypertension and TOD was not only attributed to nocturnal hypertension or morning surge in blood pressure, but to some certain own factors. For example, morning hypertension may reflect inadequate antihypertensive treatment (7) or increased activities of neurohumoral factors in the morning, such as the sympathetic nervous system and renin–angiotensin system, which contribute to progression of arterial damage (31, 32).

Based on the results of our study and others, morning hypertension is believed to provide reliable information on BP control and high risk of TOD in CKD patients. Controlling morning BP should be considered an important measure for preventing CV and renal damage. However, current ABPM guidelines do not highlight the importance of morning BP, let alone treatment of morning hypertension (6, 33). Treatment of morning hypertension may not have attracted sufficient attention because of the absence of outcome trial evidence on its benefits. Only one interventional study demonstrated that morning BP control is associated with LVH resolution and can delay the progression of CKD (34). Additional prospective studies are necessary.

Our study had some strengths. First, to our knowledge, this is the first study to investigate the correlation between morning hypertension and TOD in CKD patients. Second, compared with previous studies that measured home morning BP (7, 9, 10, 14, 15), ABPM provided readings of nocturnal BP and morning surge in blood pressure, and additional readings of morning BP, and the influence of nocturnal hypertension and morning blood pressure surge on the association between morning hypertension and TOD was clarified. This study also had limitations. This was a cross-sectional study, and the causal association between morning hypertension and TOD could not be validated. Additionally, the sample size in this study (447) was relatively small. Finally, some important data including history of sleep apnea, and socioeconomic status were missing in this study; these data need to be complemented in the future research.

In conclusion, morning hypertension was strongly correlated with TOD, including LVH, higher UPCR, LVMI, and lower level of eGFR in CKD patients with hypertension, independent of nocturnal hypertension and morning surge in blood pressure. Morning hypertension in CKD patients warrants further attention. Proper management of morning BP may reduce cardiorenal injury in these patients.

## Data Availability Statement

The original contributions presented in the study are included in the article/Supplementary Material, further inquiries can be directed to the corresponding author/s.

## Ethics Statement

The studies involving human participants were reviewed and approved by West China Hospital, Sichuan University. The patients/participants provided their written informed consent to participate in this study.

## Author Contributions

XL, FL, TZ, ZZ, HZ, AQ, YT, and WQ: conception, design, analysis, and interpretation of data. XL and FL: drafting the article or revising it. XL, YT, and WQ: providing intellectual content of critical importance to the work described. XL, FL, TZ, ZZ, HZ, AQ, YT, and WQ: final approval of the version to be published. All authors contributed to the article and approved the submitted version.

## Conflict of Interest

The authors declare that the research was conducted in the absence of any commercial or financial relationships that could be construed as a potential conflict of interest.

## Publisher's Note

All claims expressed in this article are solely those of the authors and do not necessarily represent those of their affiliated organizations, or those of the publisher, the editors and the reviewers. Any product that may be evaluated in this article, or claim that may be made by its manufacturer, is not guaranteed or endorsed by the publisher.
