# Supplementary material for: The Association of Morning Hypertension With Target Organ Damage in Patients With Chronic Kidney Disease and Hypertension
Source: Front Cardiovasc Med. 2021 Aug 26;8:715491. doi: 10.3389/fcvm.2021.715491 (PMC8427187; doi:10.3389/fcvm.2021.715491)
Supplement: Supplementary file 1 [file Data_Sheet_1.docx]

Supplemental Table 1. univariate analysis for association of morning hypertension with target organ damage.

| variables | Morning normotension  (n= 253) | Morning hypertension  (n= 194) | P |
| --- | --- | --- | --- |
| Lg LVMI | 1.93 (0.10) | 1.99 (0.10) | P<0.001 |
| Lg PCR | -1.45 (0.58) | -1.09 (0.71) | P<0.001 |
| $\sqrt{eGFR}$ | 6.98 (1.79) | 6.40 (2.07) | 0.002 |
| LVH | 54 (21.3) | 80 (41.2) | P<0.001 |

Abbreviations. LVMI: left ventricular mass index; PCR: protein-creatinine ratio; eGFR: estimated glomerular filtration rate; LVH: left ventricular hypertrophy.

Supplemental Table 2. univariate analysis for association of night hypertension with target organ damage.

| variables | Night normotension  (n=127) | Night hypertension  (n= 320) |  |
| --- | --- | --- | --- |
| Lg LVMI | 1.91 (0.09) | 1.97 (0.11) | P<0.001 |
| Lg PCR | -1.53 (0.50) | -1.19 (0.69) | P<0.001 |
| $\sqrt{eGFR}$ | 7.13 (1.46) | 6.57 (2.07) | 0.002 |
| LVH | 22 (17.3) | 112 (35) | P<0.001 |

Abbreviations. LVMI: left ventricular mass index; PCR: protein-creatinine ratio; eGFR: estimated glomerular filtration rate; LVH: left ventricular hypertrophy.

Supplemental table 3. univariate analysis for association of morning surge with target organ damage.

| variables | Low morning surge 1 (n=295) | High morning surge 2 (n=152) |  |
| --- | --- | --- | --- |
| Lg LVMI | 1.95 (0.11) | 1.95 (0.10) | NS |
| Lg PCR | -1.35 (0.62) | -1.17 (0.72) | 0.026 |
| $\sqrt{eGFR}$ | 6.72 (1.99) | 6.75 (1.82) | NS |
| LVH | 85 (28.8) | 49 (32.2) | NS |

Abbreviations. LVMI: left ventricular mass index; PCR: protein-creatinine ratio; eGFR: estimated glomerular filtration rate; LVH: left ventricular hypertro

Supplemental table 4. Association of morning systolic blood pressure, diastolic blood pressure (per 10mmHg) with target organ damage.

|  | Model 1 | Model 2 | Model 3 | Model 4 |
| --- | --- | --- | --- | --- |
| $\sqrt{eGFR}$ |  |  |  |  |
| Morning SBP (per 10mmHg) | -0.20 (-0.29, -0.11) ^*^ | -0.22 (-0.33, -0.11) ^*^ | -0.21 (-0.32, -0.10) ^*^ | -0.14 (-0.24, -0.05) ^#^ |
| Morning DBP (per 10mmHg) | -0.06 (-0.19, 0.07) | -0.01 (-0.15, 0.14) | -0.14 (-0.30, 0.02) | -0.23 (-0.37, -0.10) ^*^ |
| Lg LVMI |  |  |  |  |
| Morning SBP (per 10mmHg) | 0.018 (0.013, 0.022) ^*^ | 0.018 (0.012, 0.024) ^*^ | 0.019 (0.013 0.025) ^*^ | 0.016 (0.01, 0.022) ^*^ |
| Morning DBP (per 10mmHg) | 0.019 (0.012, 0.026) ^*^ | 0.017 (0.009, 0.024) ^*^ | 0.017 (0.008, 0.025) ^*^ | 0.016 (0.007, 0.024) ^*^ |
| Lg PCR |  |  |  |  |
| Morning SBP (per 10mmHg) | 0.11 (0.07, 0.14) ^*^ | 0.08 (0.04, 0.12) ^*^ | 0.10 (0.06, 0.13) ^*^ | 0.04 (0.01, 0.08) ^a^ |
| Morning DBP (per 10mmHg) | 0.14 (0.10, 0.19) ^*^ | 0.11 (0.06, 0.16) ^*^ | 0.10 (0.04, 0.15) ^*^ | 0.05 (0.0, 0.10) ^a^ |
| LVH |  |  |  |  |
| Morning SBP (per 10mmHg) | 1.34 (1.20, 1.50) ^*^ | 1.31 (1.15, 1.50) ^*^ | 1.36 (1.18, 1.58) ^*^ | 1.32 (1.12, 1.55) ^*^ |
| Morning DBP (per 10mmHg) | 1.34 (1.16, 1.55) ^*^ | 1.25 (1.06, 1.47) ^#^ | 1.33 (1.10, 1.61) ^#^ | 1.35 (1.10, 1.66) ^#^ |

* p< 0.001; # p<0.01; ^a^ P < 0.05

Model 1: morning hypertension

Model 2: model 1+night hypertension+ morning surge

Model 3: model 2+age, sex, BMI, alcohol, smoking, CV history, DM

Model 4: model 3+hemoglobin, albumin, Na, k, TG, office BP control for eGFR; hemoglobin, albumin, sodium, kalium, creatinine, uric acid, triglyceride, office BP control for Lg LVMI, hemoglobin, albumin, glucose, p*ca, creatinine, cholesterol, high density lipoprotein, low density lipoprotein, office BP control for Lg PCR.; hemoglobin, albumin, Creatinine, sodium, kalium, triglyceride, high density lipoprotein, low density lipoprotein, office BP control for LVH.

Abbreviations. SBP: systolic blood pressure; DBP: diastolic blood pressure; BP: blood pressure; LVMI: left ventricular mass index; PCR: protein-creatinine ratio; eGFR: estimated glomerular filtration rate; LVH: left ventricular hypertrophy.
